# A remarkable new genus and a new species of chewing louse (Phthiraptera, Ischnocera, Philopteridae) from Brazil

**DOI:** 10.3897/zookeys.541.6022

**Published:** 2015-12-01

**Authors:** Michel P. Valim, Armando C. Cicchino

**Affiliations:** 1 Museu de Zoologia, Universidade de São Paulo, Av. Nazaré, 481, Ipiranga, São Paulo, SP 04263-000, Brazil; 2 Laboratorio de Artrópodos, INBIOTEC-CONICET-UNMdP, Universidad Nacional de Mar del Plata, Funes 3300, 7600 Mar del Plata, Buenos Aires Province, Argentina

**Keywords:** *Brueelia*-complex, *Bobdalgleishia*, Jacamar, Galbulidae, Ischnocera, new genus, new species, Neotropical

## Abstract

A new genus of chewing louse as *Bobdalgleishia*, and its type species *Bobdalgleishia stephanophallus* **sp. n.** (Phthiraptera) belonging to the *Brueelia*-complex (Ischnocera: Philopteridae) are described. Adults of the new species are fully described, illustrated and compared morphologically with the type species of *Motmotnirmus* Mey & Barker, 2014, which is its closest relative. The type host of *Bobdalgleishia stephanophallus* is a subspecies of the great jacamar *Jacamerops aureus ridgwayi* Todd, 1943, an endemic Amazonian bird distributed in northern Brazil, and the type locality is the State of Pará. *Bobdalgleishia* is a remarkable genus with unique morphological and chaetotaxic characters which readily separate it from other members of the *Brueelia*-complex, in particular by having the first two marginal temporal and ocular setae very long.

## Introduction

The known chewing lice of the family Philopteridae (Ischnocera) parasitic on Galbuliformes (Aves) are six species of the genus *Mayriphilopterus* Mey, 2004 (*Philopterus*-complex) (Mey 2004, Valim and Linardi 2007) and eight species of the genus *Picicola* Clay & Meinertzhagen, 1938 (*Degeeriella*-complex) (Oniki and Emerson 1981, Valim and Linardi 2006, Price and Weckstein 2006). In addition, there is an unconfirmed record of the menoponid *Menacanthus caudatus* (Giebel, 1876) (Amblycera, Menoponidae) on *Galbula ruficauda* Cuvier (Galbulidae) (Giebel 1876, Price et al. 2003). However, there is no previous record of chewing lice from the great jacamar, *Jacamerops aureus* (Statius-Müller, 1776) (Price et al. 2003, Price and Weckstein 2006).

Among the genera belonging to the *Brueelia*-complex (Philopteridae *sensu lato*), some have a worldwide distribution, while others are geographically endemic and/or with host distribution restricted to certain host group (Mey and Barker 2014, Valim and Palma 2015). Six named genera of the *Brueelia*-complex contain species endemic to the Neotropical Region: *Bizarrifrons* Eichler, 1938 on Icteridae (Aves: Passeriformes: Passeri); *Formicaphagus* Carriker, 1957 on Thamnophilidae, Conopophagidae and Formicariidae (Aves: Passeriformes: Tyranni); *Formicaricola* Carriker, 1957 on Formicariidae; *Pseudocophorus* Carriker, 1940 on Cotingidae (Aves: Passeriformes: Tyranni); *Motmotnirmus* Mey & Barker, 2014 on Momotidae (Aves: Coraciiformes); and *Paragoniocotes* Cummings, 1916 on Psittacidae (Aves: Psittaciformes). At least four other louse genera, with species parasitic on several groups of avian hosts (e.g. Passeriformes, Trogoniformes, Piciformes), also occur in the Neotropical Region, but are not restricted to that region (Mey and Barker 2014). Our aim is to provide a detailed morphological description of a seventh endemic genus within the *Brueelia*-complex from the neotropics. The new genus is distinct from its sympatric relatives, as well as from all other genera included in the *Brueelia*-complex, both by morphological and chaetotaxic characters in both sexes. This is the first record of a member of the *Brueelia*-complex on Galbuliformes hosts.

## Methods

The specimens examined for the descriptions of the new taxa were collected from a bird skin held at the Museu Nacional do Rio de Janeiro (MNRJ), as recommended by Mey (2002). All lice collected were in good conditions and were permanently slide-mounted using Canadian balsam, as described by Palma (1978). They are deposited in the Phthiraptera collection of the Museu de Zoologia da Universidade de São Paulo (MZUSP). The geographic coordinates given for the locality of the skins searched for lice were taken from Paynter and Traylor (1991).

The nomenclature of louse head features and setae follows Clay (1951) and Mey (1994); the occipital head sensilla (*s1*–*s5*) are named following Valim and Silveira (2014). Abdominal chaetotaxy patterns are described following those in Cicchino and Castro (1996) and Cicchino and Valim (2008) for members of the *Brueelia*-complex. The classification and nomenclature of hosts follow Dickinson (2003).

Abbreviations used for both body and head setae and sensilla are given in italic and lower case (see Clay 1951, Mey 1994, Valim and Silveira 2014): ads = anterior dorsal seta, as = anterior seta, avs = anterior ventral seta, dsms = dorsal submarginal seta, mds = mandibular seta, mts = marginal temporal seta, os = ocular seta, pas = preantennal seta, pcs = preconal seta, pns = postnodal seta, pos = preocular seta, ppss = prothoracic postspiracular seta, pts = postemporal seta, s = spine-like seta of pterothorax, s1–s5 = occipital head sensilla, tr = trichoid setae, vsms = ventral submarginal seta.

Images were taken using a Leica DFC295 digital camera installed at a Leica DM5000 B optical microscope, and measurements of specimens were taken using the software Leica Application Suite (LAS) v.4.1.0. Measurements are in millimeters, and identified by the following abbreviations: ANW anterior notch width (from tips of marginal carina), PAW preantennal width (at level of *pas*), TW temple width (at level just anterior to *mts1*), HL head length (at midline including the hyaline margin), PW prothorax width (at the widest point), PL prothorax length (at midline), PEW pterothorax width (at the posterior level), PEL pterothorax length (at midline), AWV abdomen width on segment V, AL abdomen length, BAW basal apodeme width (at the widest point), BPW basal plate width, MEW mesosoma width (at the widest point), PRW penial ring width (breadth of gonopore opening), PAW paramere width (at its mid length), BAL basal apodeme length, MEL mesosoma length (including the tip of gonopore), PAL paramere length, BAMEL = BAL+MEL, GL total genitalia length (from proximal tip of basal apodema to distal tip of paramere), TL total body length (from hyaline margin of head to end of tergite XI).

## Taxonomic treatment

### Phthiraptera Haeckel, 1896
Ischnocera Kellogg, 1896
Philopteridae Burmeister, 1838 (*sensu lato*) The *Brueelia*-complex

Currently, this complex comprises 17 named genus-group taxa. A full account of the morphology and discussions on the genera included in this complex can be found in Mey and Barker (2014) and in Valim and Palma (2015).

#### Bobdalgleishia

Taxon classificationAnimaliaPhthirapteraPhilopteridae

Valim & Cicchino gen. n.

http://zoobank.org/2F42B28A-D727-4E6C-8F11-44E882AFFD90

##### Type species.

*Bobdalgleishia stephanophallus* Valim & Cicchino, sp. n.

##### Diagnosis.

*Bobdalgleishia* is morphologically close to *Motmotnirmus* (from Momotidae hosts), being the only members of the *Brueelia*-complex with *mts2* very long (subequal to *mts3*) on the temporal margin, and with sternal segments II–VI lacking sclerotization and with more than one pair of setae. All other genera of this complex have only the *mts3* very long and the sternites usually bear one pair of setae each. However, both sexes of *Bobdalgleishia* can be distinguished from those of *Motmotnirmus*, as well as from those of all other genera of the *Brueelia*-complex, by having *os* and *mts1*–*3* very long, and postspiracular present on segment II. In both sexes of *Motmotnirmus* only *mts2* and *mts3* are very long, the *os* and *mts1* are very short (see Fig. 3D), and postspiracular seta is absent on tergite II. Furthermore, males are different in the shape of the parameres (triangular and flattened in *Motmotnirmus*), by a tubiform mesosome (short and compact in *Motmotnirmus*, see Fig. 3E), and a gonopore with a distinct crown with indentations (gonopore without crown in *Motmotnirmus*, see Fig. 3E). In females, tergites XI are fused with IX+X (in *Motmotnirmus* they are separated), and each gonaphophysis has only one spine-like seta (Fic. 3C) (a patch of 3–4 thin setae in *Motmotnirmus*, as in Fig. 3F). Females of species of *Paragoniocotes* also have setae on the gonapophyses, but more than one each side, in addition to having *os* and *mts1*–*2* very short. Lastly, a very long ocular seta (*os*) is also found in one species of *Rostrinirmus* Złotorzycka, 1964 (not recognized as valid by Price et al. 2003, Valim and Palma 2015) but, as in most other members of the *Brueelia*-complex, they have very short *mts1*–*2*.

*Bobdalgleishia* is distinct from the type species of *Brueelia* *sensu stricto* at first glance by (1) the *as2*, *as3* and *dsms* set on the hyaline margin, not on the sclerotised portion of the head; (2) presence of *as3*; (3) *os* and *mts1–2* are macrochaeta. Furthermore, the type species of *Bobdalgleishia* is the only member of the *Brueelia*-complex having, in both sexes, (1) four long setae on the temple margin: *os* and *mts1*–*3*; (2) *pos* short and set on eye lens; (3) one pair of anterior setae on tergite II; and (4) sterna II–VI with more than one pair of setae and lacking sclerotized plates. In addition, females lack the cross-piece on the vulvar margin, and their tergite XI is fused with IX+X.

##### Description.

**Figure 1.**
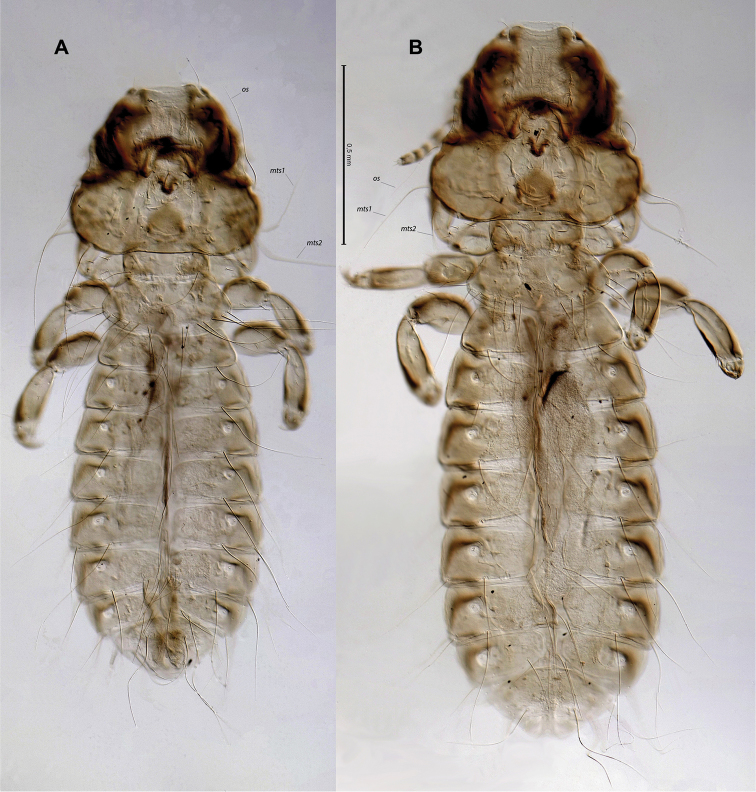
*Bobdalgleishia stephanophallus*, habitus in dorsal view: **A** male **B** female. Abbreviations: *mts*, marginal temporal setae 1 and 2; *os*, ocular seta.

**Figure 2.**
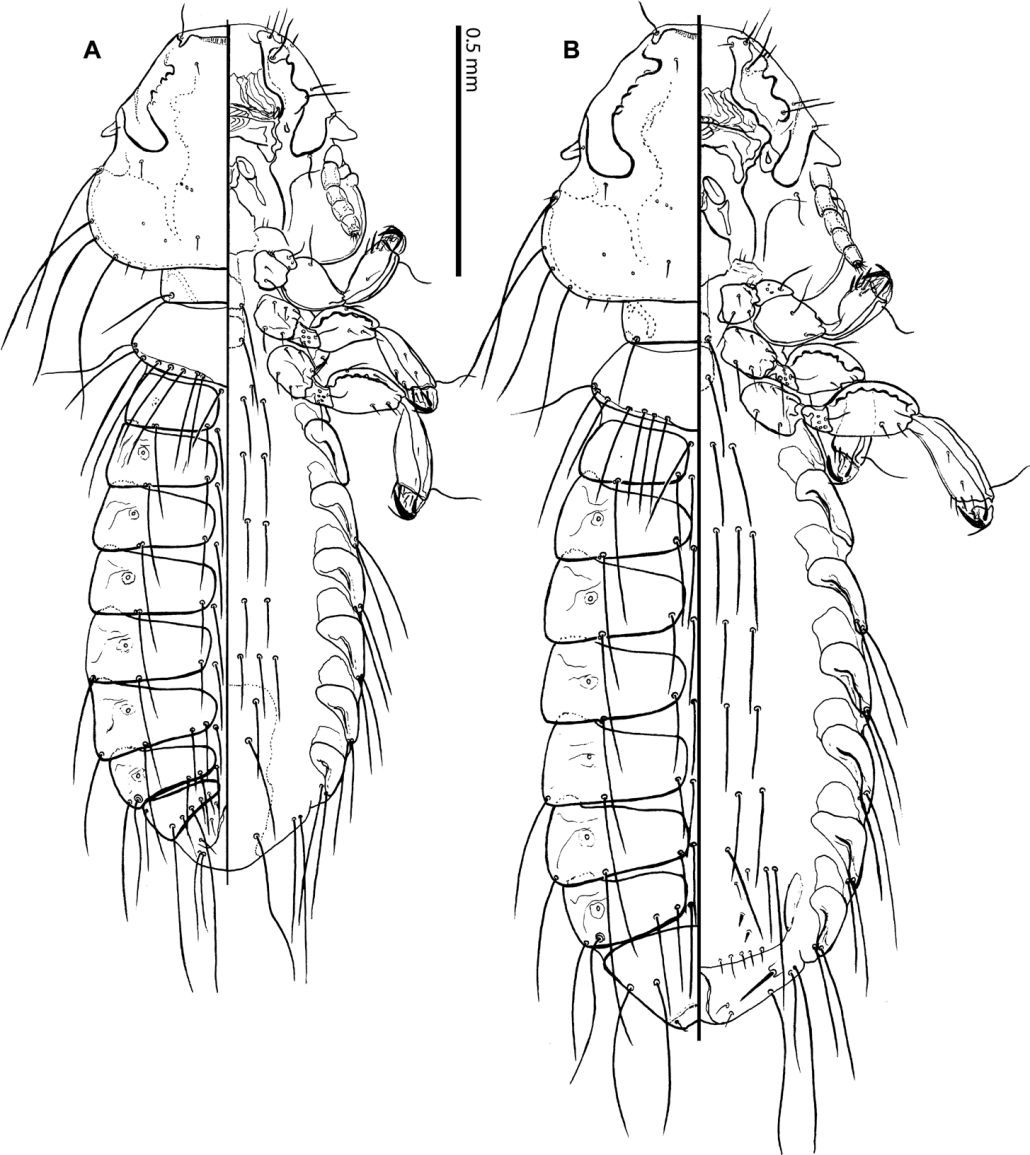
*Bobdalgleishia stephanophallus*, habitus in dorsal-ventral views: **A** male **B** female.

**Figure 3.**
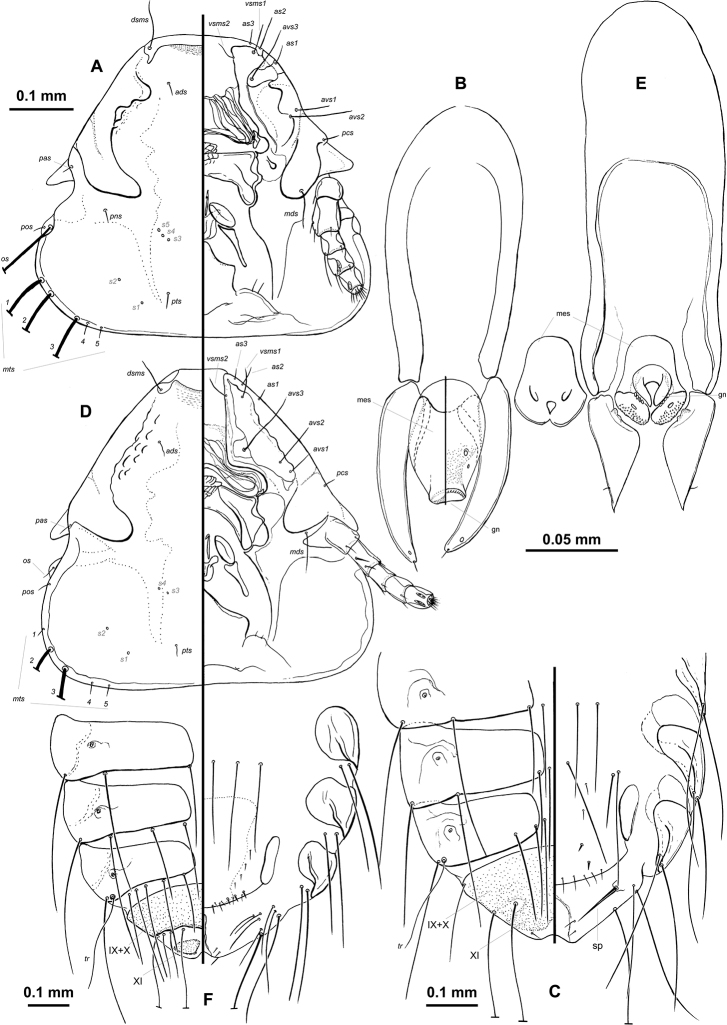
*Bobdalgleishia stephanophallus*: **A** male head in dorso-vental views **B** male genitalia, mesosome in dorso-ventral views **C** female terminalia (VI–XI), in dorso-ventral views. *Motmotnirmus marginellus* **D** male head in dorso-ventral views **E** male genitalia, mesosome in ventral view, detail of mesosomal plate in dorsal view **F** female terminalia (VI–XI), in dorso-ventral views. Abbreviations: gn, gonopore; mes, mesosome; sp, spine-like seta of the gonapophyses; IX, X, XI, last three female abdominal tergites (9^th^ to 11^th^) [for other abbreviations, see methods].

**Both sexes.** *Head*: Antennal scape and flagellomeres not enlarged; preantennal region short and tapered, conspicuously symmetric (Figs 1–2, 3A) and antennae monomorphic; anterior dorsal head plate indicated only by its anterolateral angles, but basically fused on its lateral and posterior portions with the head’s roof. Marginal carina medially divided and without lateral interruption; hyaline margin present, reaching only the distal portion of anterior dorsal head plate (ADHP), and set between anterior setae (*as2*) and widely anterior to ADHP. Dorsal setae: *ads* short and set on dorsal sclerotized surface; *dsms* medium-long and arising from preantennal suture which divides the marginal carina with discrete lateral division. Except for *dsms* medium-long, all other anterior setae short. Anterior setae *2* (*as2*) and *as3* present and set on hyaline margin. Ventral anterior head plate indistinct, ventral carina interrupted medially and fused anteriorly on each side with the marginal carina; each half of the ventral carina entirely sclerotized and with flattened lobes to attachment of pulvinus. Ocular setae (*os*) very long, *pos* very short and set more ventrally on ocular lens. Temporal carina not developed and postantennal region without sutures; both postnodal seta (*pns*) and post-temporal seta (*pts*) present and short, but never sensilliform. Head sensilla present (*s1*–*s5*), each bearing a much reduced seta, and *s5* set closer to *s3*–*s4* than to *pns.* Presence of *s5* is not regular and bilateral in all studied specimens, it maybe a duplication of *s4*. Marginal temporal setae 1–3 (*mts1*–*3*) very long, and *mts4*–*5* very short (Fig. 3A). Occipital carina present, weakly sclerotized. Gular plate roughly rhombic in shape and well sclerotized (Figs 1–2, 3A).

*Thorax*: As in Figs 1–2. Prothorax roughly rectangular, with rounded lateral sides and posterior margin nearly straight, with one pair of long prothoracic postspiracular setae (*ppss*). Pterothorax without signs of division between meso- and metathorax, with one ventral spine-like, and one dorsal trichoid, and circa of seven setae on each side of its posterior margin. Legs without distinctive features, except for the thickened dorsal incrassation of the legs II and III, thicker on femurs and with irregular inner border.

*Abdomen*: Similar in both sexes (Figs 1–2); tergite II (actually I+II) with one long pair of anterior setae (reminiscent of those from tergite I); postspiracular setae present on II–VII; without accessory to postspiracular seta; pleural setae present on IV–VIII; and sutural and innermost setae present each side on tergites II–VII. Sternites II–VI with more than two pairs of setae each. Porotaxy: sensilla present on tergites II–V.

**Male.** Antennal scape and flageromeres not enlarged, as in females. Subgenital plate faintly delimited and with two pairs of setae at level of sternite VII. Tergal plates IX+X fused, distinct and medially divided, tergite XI indistinct or non-sclerotized. Genitalia as in Fig. 3B, see detailed description below.

**Female.** Subgenital plate smooth (Fig. 3C), lacking any sclerotization on distal vulvar margin (the “cross piece” of Ansari 1956), posterior end nearly rectangular; with three pairs of setae at level of sternite VII. Tergites XI fused with IX+X, forming a single IX–XI last segment (Figs 2B, 3C).

##### Etymology.

Named in honor to the late and personal friend Robert [Bob] C. Dalgleish (1940–2009) for his special disposition to listen and learn from those who disagree with him on taxonomic issues. Bob was an example of how a taxonomist might make a huge contribution in a relatively short period of time, less than ten years in his second life period of “lousying” with us (his first was during 1966–1972). It is a noun in the singular genitive, masculine.

#### Bobdalgleishia stephanophallus

Taxon classificationAnimaliaPhthirapteraPhilopteridae

Valim & Cicchino sp. n.

http://zoobank.org/E28B4C23-47CD-437D-AC57-D97ECB650CFB

Figs 1 , 2 , 3A–C

##### Type host.

*Jacamerops aureus ridgwayi* Todd, 1943 – great jacamar [ridgwayi] (Galbulidae).

##### Type locality.

Alto Rio Cururu, Pará, Brazil.

##### Diagnosis.

*Bobdalgleishia stephanophallus* can be easily separated from the four species of the genus *Motmotnirmus* (*Motmotnirmus marginellus* (Nitzsch [in Giebel], 1866) the type species; *Motmotnirmus xilitla* (Carriker, 1954); *Motmotnirmus guatemalensis* (Dalgleish, 1971), and *Motmotnirmus humphreyi* (Oniki & Emerson, 1982)) by the generic characters discussed above, i.e. head chaetotaxy (compare Figs 3A and 3D), male genitalia (compare Figs 3B and 3E), and female gonapophysis (compare Figs 3C and 3F). In addition, tergites VII–VIII in species of *Motmotnirmus* have more than four posterior tergal setae on each segment (Fig. 3F), in *Bobdalgleishia stephanophallus* these same segments have fewer setae (males sometimes with 1+1 on VII only) (Fig. 3C).

##### Description.

**Male.** Habitus as in Figs 1A and 2A. Body pigmentation light-yellow, except for the head marginal carina and pre-antennal nodi strongly brownish (Fig. 1A).

*Head* as in Figs 1A, 2A and 3A, slightly shorter than wide, with cephalic index (HL/TW) 0.9. Coni well developed and subequal in length with scape. Preantennal region tapered, preantennal margins slightly convex, and marginal temporal margins rounded. Small and nearly convex hyaline margin between tips of the pre-marginal carina each side (Fig. 3A). Preantennal region with internal margins of carinae distinctly thick and irregular (Figs 1A and 3A). Frontoclypeal suture light and distinct, its nodal area (preantennal nodus) roughly circular in shape and very well sclerotized. Gular plate roughly rhomboid and uniformly pigmented. Temples rounded; marginal temporal carina darker pigmented and medium thick, with its inner margin nearly uniform up to the level of *mts4* (Fig. 3A).

*Thorax* as in Figs 1A and 2A. Pterothorax with 7 marginal setae on each side (rarely 6 in one or both sides); pterothoracic apodeme (metepisternum) not well pigmented, reaching the lateral margins of the segment. Meso- and metasternal plates not fused, both grossly rounded and bearing a pair of long setae each.

*Abdomen* as in Figs 1A and 2A. Tergites uniformly pigmented, except for a small area around spiracles (Fig. 1A). Tergal chaetotaxy: postspiracular long on II–VII; accessory setae absent; and one medium long sutural seta on II–VII. Tergite VIII: trichoid lateral setae thin and medium long, and five setae subequal in length to trichoid setae. Tergite IX+X medially divided, with 2 medium long and 3–4 short setae. Paratergal chaetotaxy: II–III 0; IV–V 2; VI–VIII 3. Paratergites II–VIII with internal incrassation forming an inverted-L on each side of the abdominal segments. Sterna II–VI lacking sclerotized plates, each with four long setae (rarely 2 setae on II, or 6 on VI) set on the soft tegument, one unpaired small and anterior setae on segment II in the holotype. Subgenital plate present and sclerotized, the only sternite visible, but outline completely indistinct (Fig. 2A).

*Genitalia* as in Fig. 3B. Basal plate proximally wide, narrowing distally, with enlarged thickened lateral margins; parameres allantoid (“sausage-shaped”), their bases without defined head, but completely articulated with basal plate, each bearing one subapical sensillum and one apical microseta; mesosomal complex tube-shape, with 2 ventral pairs of sensilla each side, and distally reaching the mid-length of parameres; gonopore is also a large tube, but narrower than the mesosomal tube, and with a distinct crown bordered with indentations, more conspicuous ventrally (Fig. 3A).

Measurements (n = 2): ANW 0.10; PAW 0.39–0.40; TW 0.51–0.53; HL 0.45–0.47; PW 0.24–0.25; PL 0.13–0.14; PEW 0.35–0.36; PEL 0.13–0.15; AWV 0.51–0.54; AL 0.98–1.07; BAW 0.07–0.09; BPW 0.05–0.07; MEW 0.05; PRW 0.02–0.03; PAW 0.02; BAL 0.16; MEL 0.08–0.09; PAL 0.11–0.12; BAMEL 0.24–0.25; GL 0.26–0.28; TL 1.64–1.70.

**Female.** Habitus and coloration similar to males (Figs 1B and 2B), except for size and details of terminal segments. Head short, with cephalic index (HL/TW) 0.8. Abdominal tergites II–VII and sternites II–VI as in male for coloration, incrassation, and chaetotaxy.

*Pterothorax* with 6+5 (11 in total) marginal setae on each side. Tergites II–VIII divided medially, with internal end nearly rounded. Paratergal chaetotaxy: II–III 0; IV–V 2; VI–VIII 3. Sternal plates as in males (Fig. 2B); number of sternal setae on II 5, III 8, IV 4, V 4, VI 5. Tergite VIII: each side with one thin trichoid lateral seta, one innermost seta and one sutural seta (Fig. 3C). Tergites XI fused with those of IX+X (Figs 1B, 2B, and 3C). Morphology and chaetotaxy of terminalia as in Fig. 3C.

*Subgenital plate* indistinct in the single female studied, with 2–3 small setae on each side (Fig. 3C). Gonapophyses bear one spine-like setae each, both directed medio-posteriorly and arising from a distinct tubercle. Vulva with only two submarginal short spiniform setae on each side, and 10 medium-long thin setae on its posterior margin (Fig. 3C). Area of the subgenital plate with one pair of long medial seta, plus two pairs of medium long setae each side, all along sternum VIII (Fig. 3C).

Measurements (n = 1): ANW 0.10; PAW 0.45; TW 0.59; HL 0.50; PW 0.27; PL 0.15; PEW 0.40; PEL 0.15; AWV 0.62; AL 1.26; TL 1.94.

##### Etymology.

The species epithet is a composite of the Greek words *Στέφανο* (*stephano*-) and *φαλλός* (-*phallus*), which mean ‘a crown’ and ‘the penis’. It makes allusion to the crowned structure on the opening of the male gonopore. It is an adjective in the nominative singular.

##### Type material.

Holotype ♂ (MZUSP #6363), ex *Jacamerops aureus ridgwayi* Todd, 1943 (#A.2880, voucher at MNRJ); BRAZIL: Pará, Alto Rio Cururu (07°12'S, 58°04'W; 50m), 6.VI.1957, H. Sick coll. **Paratypes**: 1♂, 1♀ (MZUSP #6363–6364), same data as holotype.

##### Additional material examined.

*Motmotnirmus marginellus* (Nitzsch [in Giebel], 1866): 3♂, 3♀ (MZUSP #6342–6348), ex *Momotus momota* (Linnaeus, 1766) (Aves: Coraciiformes: Momotidae) (voucher at MZUSP #98878), BRAZIL: Pará, Fazenda Fartura (09°38'04.1"S, 50°28'37.6"W, 160m), Santana do Araguaia,. VIII.2014, A. Gouvea coll.

##### Remarks.

The morphological differences between the single species of *Bobdalgleishia* and those of *Motmotnirmus* are congruent with the evolutionary history of their host groups: Galbuliformes and Coraciiformes, respectively (e.g. Livezey and Zusi 2007, Hackett et al. 2008, Yuri et al. 2013). However, it is surprising to find that the shape of the mesosomal plate and the “crowned” gonopore in the male genitalia of *Bobdalgleishia stephanophallus* are unique features among all the species of the *Brueelia*-complex. Some unrelated genera of Philopteridae – Rallicola (Aptericola) Harrison, 1915 (*Rallicola*-complex) as an example – have mesosomes with similar shape (see Clay 1972: figs 13–15), whereas the mesosome and crowned gonopore are similar to those of some members of the family Heptapsogasteridae – *Rhopaloceras almeidai* Guimarães, 1946: fig 5, as an example. We believe these similarities are the result of evolutionary convergence, and have no phylogenetic implications.

Considering that the Piciformes are also included in the same large group as Galbuliformes and Coraciiformes (e.g., Livezey and Zusi 2007, Hackett et al. 2008, Yuri et al. 2013), this new genus needs to be compared with lice of the *Brueelia*-complex found on those hosts. Species from both Picidae (see Dalgleish 1971) and Ramphastidae (see Cicchino 1983) only have the *mts3* very long (against *os*, *mts1-3* very long in *Bobdalgleishia*). In addition, species from woodpeckers belong to *Brueelia* Kéler, 1936 (*sensu stricto*), whereas those found on toucans to the genus *Traihoriella* Ansari, 1947 (D.R. Gustafsson pers. comm. 2015).

## Discussion

Although museum skins are good source of bird ectoparasites (Mey 2002), there are many examples in the literature of louse species described from the wrong hosts, which have taken great time and efforts by later taxonomists to disclose their true host-parasite relationship (e.g. Palma 1994). Although MPV examined approximately 15 skins of the great jacamar in the MNRJ and a further 21 in MZUSP, the type series of *Bobdalgleishia stephanophallus* was found by chance on just one skin collected by H. Sick. Notwithstanding the small number of lice found and the risks involved in describing new louse taxa from museum skins, we accept the record as the result of correct and natural host-louse association. These are our reasons that justify the description of the two new taxa from lice collected on a museum skin, accepting the great jacamar as the true host: (1) more than one pair of specimens were collected, all of the same species, including two males showing that their differential characters were not due to distortion or individual variation; (2) all morphological and phylogenetic relevant characters needed for a complete description are in perfect condition and perfectly visible in all specimens (see Fig. 1); (3) the number and degree of morphological differences between our new taxa and their closest relatives already described in the literature are significant enough to be worthy of publication and dissemination; (4) the male genitalia of *Bobdalgleishia stephanophallus* are unique among species of the *Brueelia*-complex; (5) considering that species of *Brueelia* *sensu lato* have been recorded from several host orders (e.g. Passeriformes, Trogoniformes, Piciformes, Coraciiformes), finding the first member of the *Brueelia*-complex on a species of Galbuliformes is not unexpected; (6) if by any chance, the great jacamar is shown not to be the true, natural host of *Bobdalgleishia stephanophallus*, our morphological description is detailed enough for this taxon to be unequivocally recognized on any other host and from any part of globe; (7) the morphological characters we used to describe the new genus *Bobdalgleishia*, will distinguish it from any genus within the family Philopteridae, regardless which host may be its correct, natural host; (8) the morphological features shared between species of *Bobdalgleishia* and *Motmotnirmus* make sense, considering that the Galbuliformes arose from the same related branch which the Coraciiformes (plus Piciformes and Trogoniformes) belong to (Livezey and Zusi 2007, Hackett et al. 2008, Yuri et al. 2013), these being three orders from which species of the *Brueelia*-complex have been recorded.

## Supplementary Material

XML Treatment for Bobdalgleishia

XML Treatment for Bobdalgleishia stephanophallus
